# No-shows among children and adolescents in public oral health service: a retrospective register-based study from Finland

**DOI:** 10.1186/s12903-025-07045-4

**Published:** 2025-10-17

**Authors:** Shweta Goswami, Nora Hiivala, Inari Mensonen, Tuomo Maisala, Minna Kaila, Battsetseg Tseveenjav

**Affiliations:** 1 Clinicum Faculty of Medicine, University of Helsinki, Haartmaninkatu 1, Helsinki, 00014 Finland; 2 Oral Health Care, City of Helsinki, Helsinki, Finland; 3 Head and Neck Center, University of Helsinki and Helsinki University Hospital, Helsinki, Finland; 4 Institute of Dentistry, University of Turku, Turku, Finland; 5 Department of Maxillofacial Surgery, Päijät- Häme Joint Authority for Health and Wellbeing, Päijät-Häme Central Hospital, Lahti, Finland

**Keywords:** Oral health services, No-shows, Missed scheduled dental appointments, Use of oral services, Children & adolescents, Retrospective register-based study

## Abstract

**Background:**

The aim of this study is to assess dental no-shows or missed scheduled dental appointments among children and adolescents in public oral health services in Helsinki, Finland, where under 18-year-olds receive subsidized oral health care.

**Methods:**

This retrospective register-based study focused under 18-year-olds in Helsinki who had dental no-shows, which was defined as any failure to arrive for a scheduled appointment without notifying, in the public oral health services from 2006 to 2020. The study utilized retrospectively collected data from the City of Helsinki’s electronic patient health information register systems, Effica (2006–2017) and Lifecare (2018–2020), which were in use during the respective periods. In the context of this study, children refer age group 0–9 years and adolescents 10–17 years.

**Results:**

A total of 2,513,376 appointments were found from patient register; 92.6% (*n* = 2,326,878) were actualized visits and 7.4% (*n* = 186,498) no-shows. Dental no-shows among children and adolescents showed decreasing trend from 9.9% to 5.8% between 2006 and 2020 except of slight increase in year 2019. Of all dental no-shows, 5.2% were registered among children and 8.6% among adolescents. Boys had more frequently no-shows than girls (*p* < 0.01). Of the study population, 5% had 21.8% of all no-shows.

**Conclusions:**

The 15-year trend analysis showed a reduction in yearly dental no-show prevalence among children and adolescents, in general. However, there is a positive correlation between age and the frequency of no-shows. There was a strong polarization of the no-show phenomenon, only 5% of the children and adolescents accounting more than one fifth of all missed appointments. This polarized group needs to be characterized, so that potential underlying causes can be studied.

## Background

Oral health care services are crucial for sustaining oral health. When a patient does not attend a scheduled visit and does not cancel it, the visit is called a no-show [1]. In oral health care, no-shows can have detrimental effects such as elevated medical expenses, income loss, inefficient utilization of healthcare personnel, reduced productivity, disruption of patient care and impaired physician-patient rapport [2, 3]. Moreover, individuals who often miss their dental appointments are more likely to have untreated caries [4]. Regular dental visits enable prevention, and early detection and treatment of dental health issues [5]. A recent study from Finland showed that children from families on long-term social assistance had more no-shows and caries lesions than others [6].

Dental avoidance leads to poor oral health and later increased utilization of public oral health services [7–10]. A Swedish study of 16–19-year-olds indicated that 13.1% of 23,522 booked dental appointments were missed. Those skipping the visit had more oral health issues, required more invasive treatments, and had previously missed and cancelled dental appointments [11].

In Finland, children and adolescents have received free public oral health services, including orthodontics since 1972 [12]. Currently, around 47% of this population avail routine check-ups with a dentist or dental hygienist in the public oral health services [13]. The periodicity of oral health check-ups and examinations is dictated by Finnish Government Decree (338/2011) [14]. The Decree states that local authorities must provide at least once an examination of oral health status and need of treatment for each family expecting their first child, and an oral health examination for each child at the ages of one to two, three to four, and five to six years, carried out by dental hygienists or nurses, when needed by dentists. Likewise, schoolchildren in the 1 st, 5th and 8th grades must be examined. Specialist oral health examinations should be conducted as necessary. At the age of 17, youth receive an invitation to book dental appointments with dental hygienists. Children and adolescents at risk of oral diseases are provided with enhanced preventive oral health care [14].

The oral health of Finnish children and adolescents has improved since the 1970 s [15]. Between 2001 and 2013, approximately 40,000 children and adolescents visited the Finnish public oral health services annually, receiving a total of 2,488,805 treatment measures. However, the overall number of treatment measures has increased in the public oral health services [16]. Another study found that a significant proportion of low dental care user children and adolescents and orthodontic heavy users were caries free, while a small number in the heavy user group exhibited a significant number of caries [17].

Despite the free of charge provision of oral health services, it is not uncommon for dental appointments to be missed or cancelled among children and adolescents. It is therefore important to evaluate the rate at which dental appointments are missed in order to avoid potential negative outcomes in the future. The objective of this study was to assess the prevalence of dental no-shows among children and adolescents under 18 years of age and describe no-show trends between 2006 and 2020.

## Methods

### Study design and setting

This retrospective register-based study was based on electronic health information systems, Effica (2006–2017) and Lifecare (2018–2020), which encompasses dental care information of all the patients treated in the public oral healthcare department of City of Helsinki. Dental appointments for children and adolescents under 18-year-olds, are booked by public health centres for check-up examinations and treatment visits as well. All these visits are registered in the health information system, also in case the individual misses the appointment without cancelling. An automatic phone message service (SMS) reminder system was introduced in 2007 to prevent no-shows.

### Data extraction

In this study, a no-show refers to missing scheduled appointment without cancelling, i.e. cancellation with pre-notification was not considered a no-show. The scheduled visits were categorized into two according to treatment codes made by dentists, dental hygienists or dental nurses. The scheduled appointments were then recoded based on later events into (1) actualized visit: if any examination or treatment code was identifiable in the records, and (2) no-show (missed appointment): if the code was 900, specific and official code for missed appointment. In this study, children referred 0–9-years-olds and adolescents 10–17-years-olds, respectively. These codes are provided by the Finnish National Institute for Health and Welfare.

### Statistics

The statistical analyses were carried out using the SPSS 28.0 software (SPSS Inc., Chicago, IL, USA). Descriptive statistics were reported. Correlations between variables individual’s age and sex, observation years of the study and frequency of no-shows were calculated using Pearson’s correlation test (r). The data analyzed was then graphed using Microsoft Excel 365.

## Results

The periodicity and oral health examinations in Helsinki, according to public information provided by the City of Helsinki in 2024 [18], are depicted in Table 1. Expecting family or first-time parents as well as all age groups of children and adolescents shown in Table 1 receive invitation letters for booking their oral health examination.

**Table 1.** Periodicity and oral health check-ups in Helsinki, Finland

	Oral health check-ups carried out by
Expecting Family or First Time Parents	Dental Nurse or Remote Appointments
1-year-olds	Dental Nurse or Remote Appointments
3-year-olds	Dental Nurse
5-year-olds	Dental Nurse or Dental Hygienist
1 st grade schoolchildren (about 7-year-olds)	Dental Hygienist
3rd grade schoolchildren (about 9-year-olds)	Dentist
5th grade schoolchildren (about 11-year-olds)	Dental Hygienist
8th grade schoolchildren (about 14-year-olds)	Dentist
17-year-olds	Dental Hygienist

**Fig. 1:**
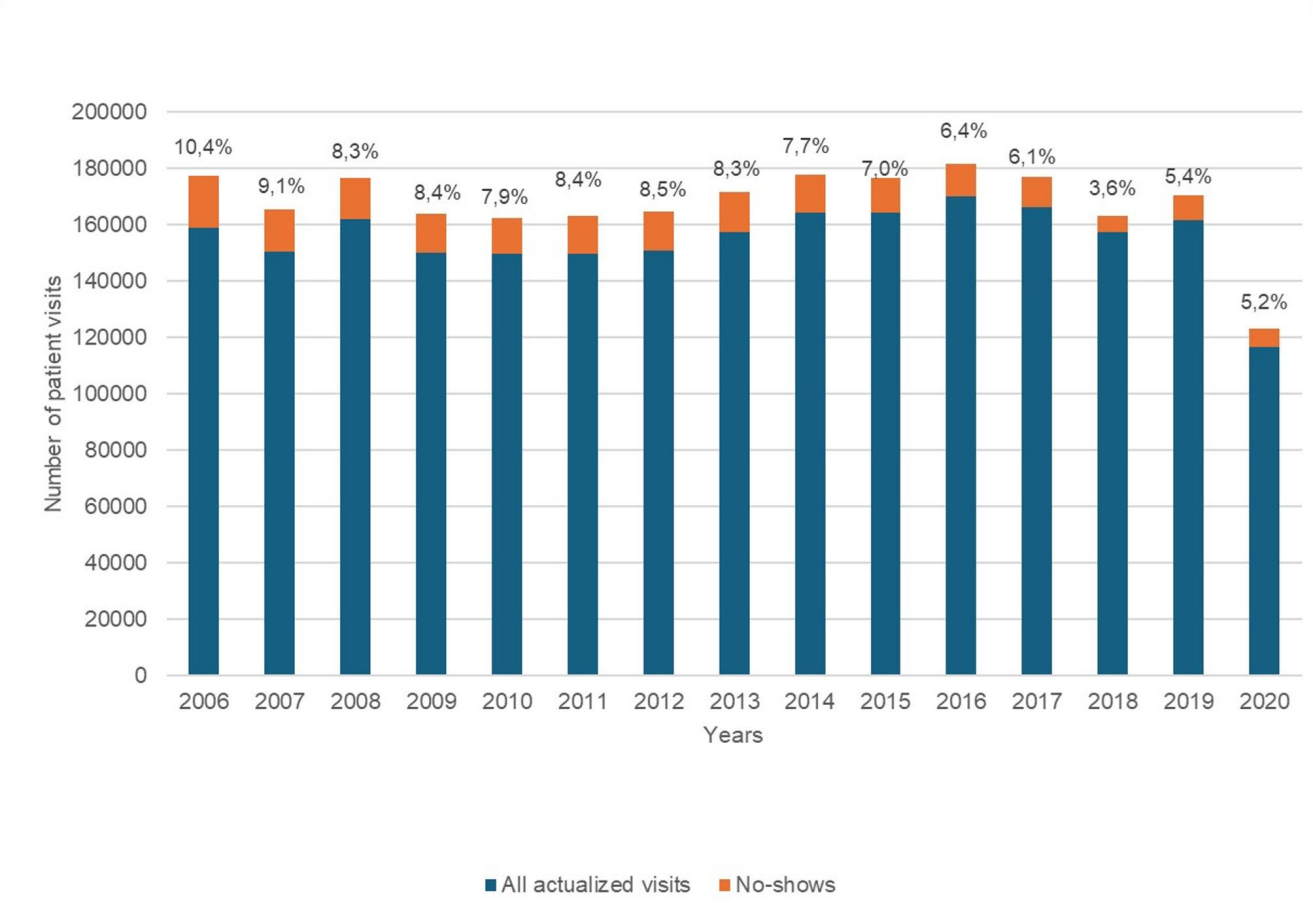
Dental actualized visits and no-shows among children and adolescents in Helsinki health centers, by years from 2006–2020

A total of 2,513,376 dental appointments were scheduled for patients aged 0–17 years from 2006 to 2020 (Fig. 1). Of these, 92.6% (*n* = 2,326,878) were actualized visits and 7.4% (*n* = 186,498) were no-shows.

**Fig. 2:**
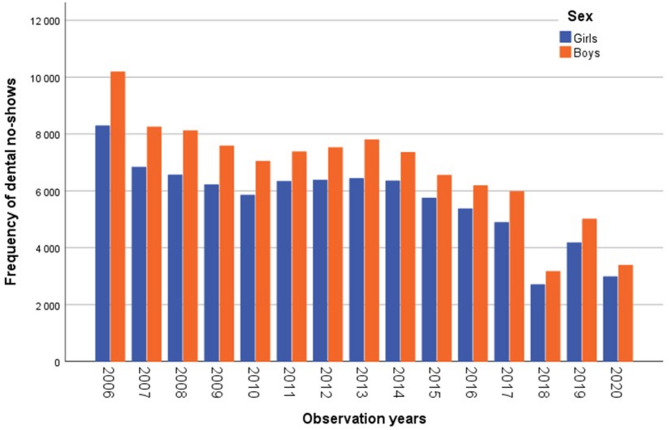
Frequency of dental no-shows by individuals’ sex and observation years in Helsinki health centers from 2006–2020

The frequency of dental no-shows declined among boys and girls from 2006 to 2020. There was total 186,497 no-shows – 85,054 among girls and 101,443 among boys. The boys accounted for 54.4% of all no-shows and surpassed the number of no-shows among girls each year. The no-shows increased between 2011 and 2013 (girls: 6,329 to 6,430; boys: 7,370 to 7,796), followed by a steady decline up to 2017. In 2020, the frequency of no-shows decreased by 64% among girls and 67% among boys compared to 2006. A significant sex difference (*p* = 0.002) in no-show rates was observed within the study period. Figure 2 shows frequency of dental no-shows by sex.

**Fig. 3:**
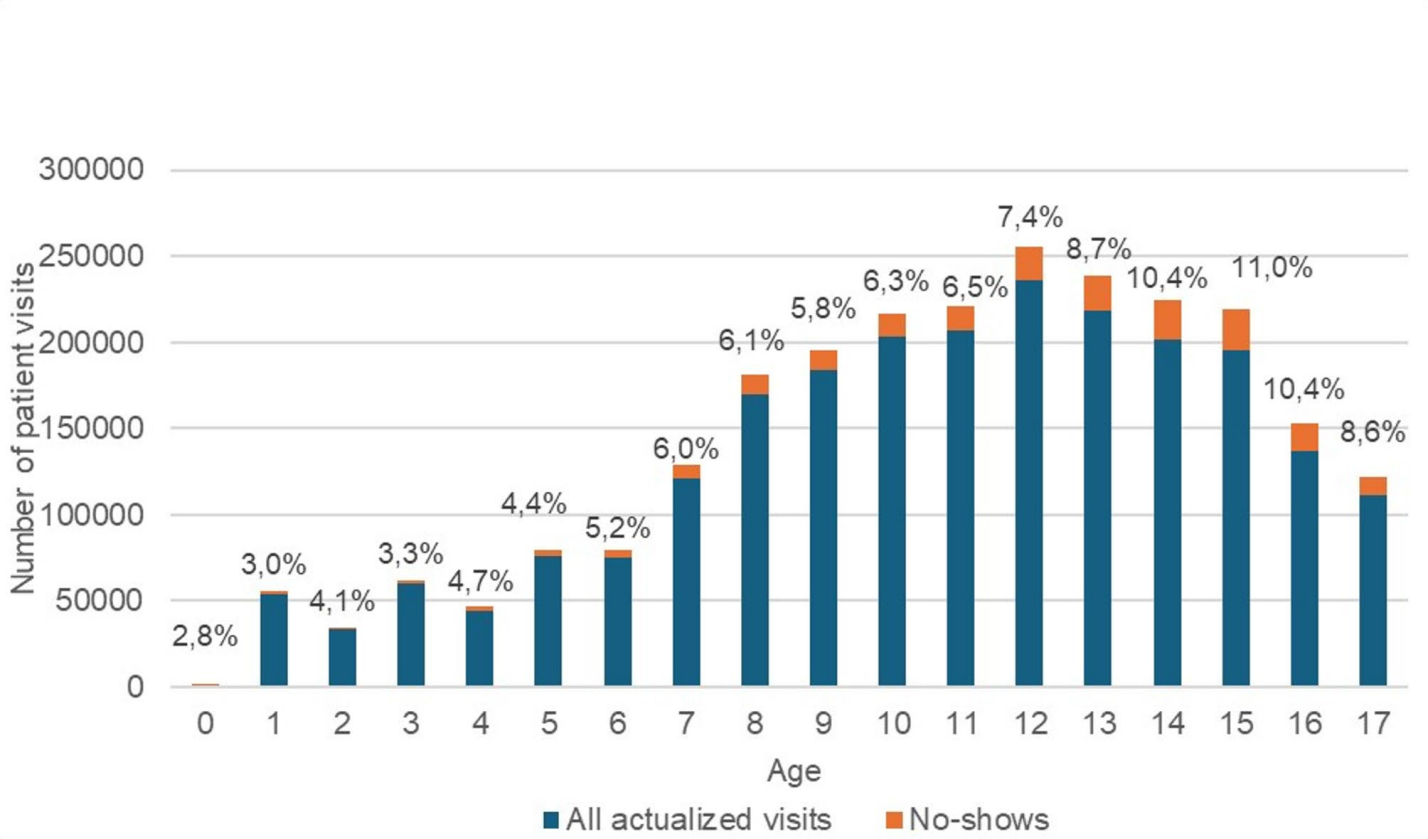
Dental actualized visits and no-shows among children and adolescents in Helsinki health centers, by their age during 2006–2020 (*n* = 2,513,376).^1^

The prevalence of no-shows was 5.2% and 8.6% among children and adolescents respectively. The non-utilization of dental health care has shown an upward trend from childhood through adolescence. The number of no-shows peaked at 14–15 years of age, where no-shows accounted for 10.7% and 11.0%, respectively (Fig. 3).

A positive correlation was found between age and the frequency of no-shows (*r* = 0.872, *p* < 0.001). However, an inverse association was seen between the number of years and frequency of no-shows (*r* = −0.884, *p* < 0.001). Among those with one or more no-shows, 53% were boys and 47% girls.

**Fig. 4:**
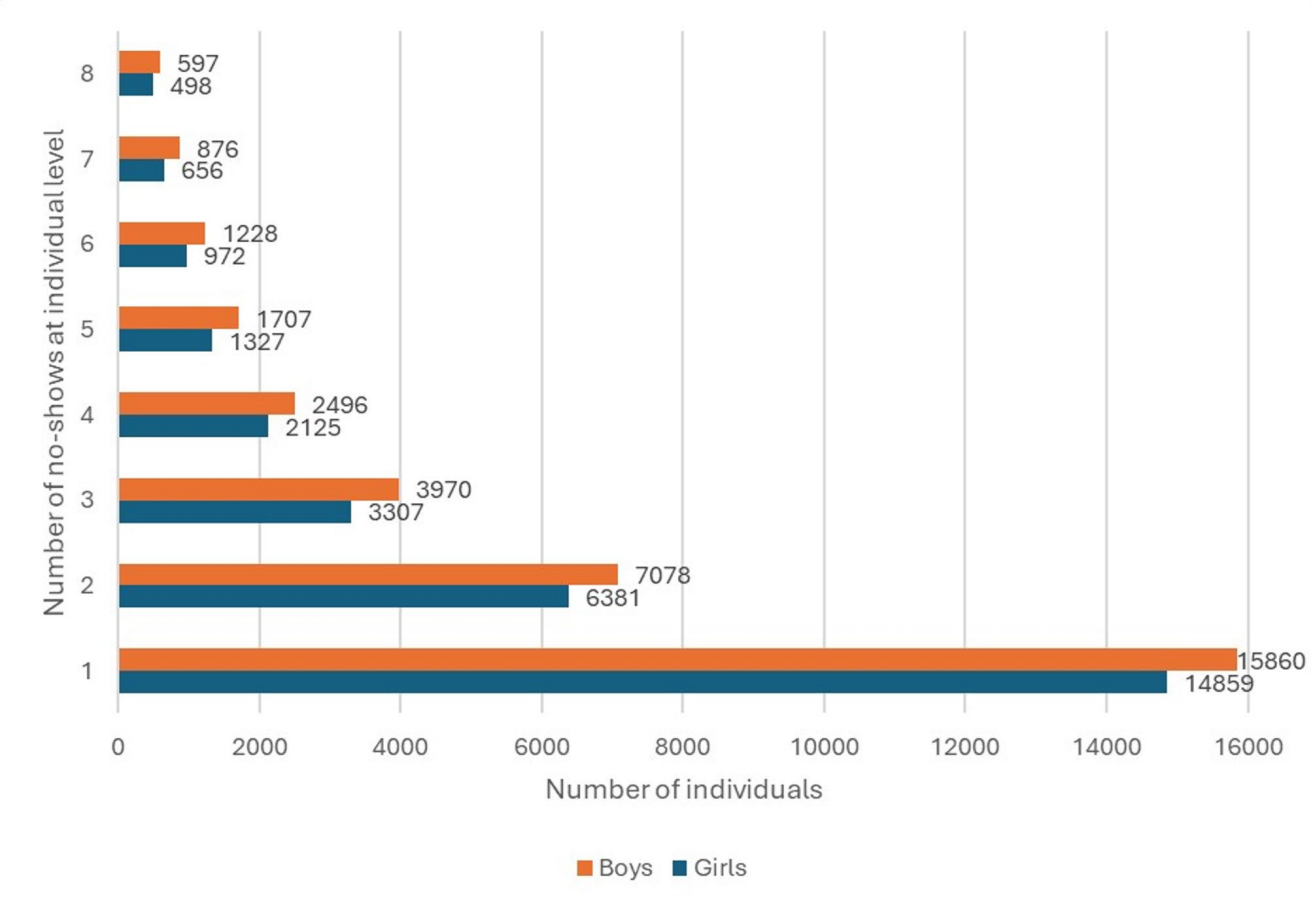
Distribution of 1–8 dental no-shows among children and adolescents at individual level, separately by sex (*n* = 145,806), in Helsinki health centers during 2006–2020

All no-shows (186,498) between 2006 and 2020 belonged to 67,284 children and adolescents. Of all, 45.7% (*n* = 30,719) of the children and adolescents had one missed appointment and 49.3% (*n* = 33,218) had two to eight missed appointments, accounting for 115,087 of the total no-shows. Fig. 4 shows children and adolescents (*n* = 63,037) with 1 to 8 no-shows; cumulative frequencies accounting 95% of all no-shows. In each scale group (Fig. 4), no-shows differed by sex (*p* > 0.05). Of all children and adolescents, 5% (*n* = 3,347) had 9 to 40 no-shows, which accounted 21.8% (*n* = 40,692) of all missed appointments, revealed a strong polarization of the phenomenon.

## Discussion

The findings of this study indicate that there is a significant positive and inverse correlation with age and observation years (*p* < 0.001) in the prevalence of dental no-shows among children and adolescents, emphasizing the credibility and robustness of these associations.

Dental no-shows were more frequent in adolescents and among boys than in children and among girls, respectively. The present decreasing trend from 2006 to 2020 differed from our previous study, which showed an increasing trend of no-shows among children and adolescents in specialized care from 2009 to 2023 [19]. This could be related to the centralization of dental general anesthesia patients in specialized care, increasing numbers of both actualized visits and no-shows [19]. According to the present and previous results [20], no-shows are more frequent among adolescents than among children.

Although free dental services are accessible among children and adolescents in Helsinki health centers, there is still a noticeable disparity in their utilization. Failing to attend scheduled visits may negatively impact the patient’s health as a result of the delay in diagnosis or treatment [1]. Of children who missed more than 10% of their dental appointments, 74% had untreated caries [4]. Missed dental appointments can lead to poorer oral health as well as exacerbated emergent dental needs [4, 21]. Children who miss their scheduled dental appointments should be classified as high-risk patients and provided with personalized treatment plans [9].

In this study, the prevalence of dental no-shows among children was 5.2%. According to a study, 15% of children who had missed at least one appointment in the previous two years had increased enamel disturbances, caries experience, and caries activity as well as longer treatment duration [9]. Children who had one or more missed dental appointments, had behavior management issues, dental anxiety, and parents who avoided dental care were more likely to have caries experience at the age of 5 years [22]. Previous research has documented the prevalence of missed dental appointments among the pediatric population ranging from 6.7% to 52% [23–27].

Among adolescents, the dental no-show rate was found to be 8.6% in this study. The findings of previous studies showed the prevalence of dental avoidance (irregular, delayed, cancelled, and no-show) has been estimated to reach 20% among adolescents in Scandinavia including Norway and Sweden where oral health care services are free of charge among children and adolescents [7, 11, 28]. Dental care during adolescence is crucial for multiple reasons, such as the emergence of permanent teeth, which leads to an increased risk of tooth decay on more surfaces, as well as a higher likelihood of early periodontal disease [29, 30]. Hence, adolescents are more susceptible to oral diseases during a crucial developmental stage while they are forming oral hygiene routines [31]. The prevalence of dental no-shows was found to be lower among individuals aged 16 and 17 compared to the uptrend pattern observed in 0–15 years old in this study. This could be attributed to the fact that the number of actualized visits was also lower among them. According to the information retrieved from the City of Helsinki, about 43% of 17-year-olds did not schedule an appointment despite receiving an invitation letter.

The results of the present study have found adolescents had a higher dental no-show rate than children. This finding is in accordance with previous studies [7, 32, 33]. By contrast, some studies report that dental no-shows tend to be higher in children than adolescents [20, 34]. This might be related to the different sociodemographic and other related characteristics [20, 34]. The disparity in the utilization of dental services is influenced by individual, social, cultural, and economic determinants [35].

A recent review has suggested various factors for non-utilization of dental services including predisposing (age, gender, ethnicity, parent’s level of education), enabling (family income, dental insurance) and treatment need factors (oral clinical status) [20]. Those belonging to racially and ethnically varied origins face a greater likelihood of having unmet dental health needs, as well as those with intricate medical conditions [36, 37]. Therefore, it is important to understand the specific patient characteristics that are connected with this behavior in order to establish focused intervention programmes [38]. The role of parents in children’s oral health is crucial. Parents can have a direct impact on the prevention of oral and other diseases of their children by teaching them healthy behaviors.

The declining trend of no-shows during the observation years of this study may partly reflect the effectiveness of various measures implemented by oral health care system to minimize the magnitude of missed appointment, such as sending SMS-reminder message to caretakers of children and to adolescents themselves.

This study also showed strong polarization identified as 5% of all children and adolescents had 21.8% of all missed appointments. This phenomenon of no-shows is also seen in the previous study of children and adolescents in specialized care [19], where almost 6% of them had nearly 19% of the no-shows. Identifying the factors of polarization may reveal why some children and adolescents missed more dental appointments than others and aid in the development of interventions to alleviate the burden of dental no-shows and associated adversities.

The significant association between dental anxiety and avoidance of dental visits is widely recognized [8, 11, 39]. Previously, dental anxiety, pain experiences, poor oral health status, and a family history of avoidance have been linked to avoidance of dental visits [28]. In the present study, the dental no-show prevalence in the age groups six, 12 and 15 were 5.2%, 7.4% and 11% respectively. . Previously, high dental fear has been reported among 12- and 15‐year‐old children as compared to the younger ones [40]. Caries-affected children between six and twelve years were more likely to report dental fear than their caries-free counterparts [40].

Missed dental appointments waste publicly funded capacity and may increase treatment costs when preventive care is not taken. No-shows disrupt patients, providers and clinical procedures that leads to further difficulty in scheduling appointments. The delay of preventive care and primary treatment may accelerate disease progression, leading to more complex and costly procedures [41].

### Strength and limitation of the study

Since the data were collected from the public health sector, which provides dental services to all children and adolescents, the findings can be considered generalizable. In addition, this study for the first time explored no-shows among children and adolescents and identified the polarization of the no-show phenomenon. The present results should be applied by improved understanding of which patients are more likely to miss their appointments and the underlying reasons for their absences, in order to effectively help resolve individual barriers [42].

A limitation of this study is that the data originates from patient heath records, meaning any documentation errors remain uncorrected, as is common in register-based study. In this study, we did not relate numbers of no-shows with total dental visits or with emergency visits of the individuals.

## Conclusion

The 15-year trend analysis showed a reduction in yearly dental no-show prevalence among children and adolescents, in general. However, there is a positive correlation between age and the frequency of no-shows. There was a strong polarization of the no-show phenomenon, only 5% of the children and adolescents accounting more than one fifth of all missed appointments. This polarized group needs to be characterized, so that potential underlying causes can be studied.

## Data Availability

The datasets used and analyzed during the current study are available from the corresponding author on reasonable request.

## Footnotes

1 Bars represent the schedule appointment and percentages indicate the proportion of no-shows within each age group

## References

[1] Marbouh D, Khaleel I, Al Shanqiti K, Al Tamimi M, Simsekler MCE, Ellahham S, et al. Evaluating the impact of patient no-shows on service quality. Risk Manag Healthc Policy. 2020;13:509–17. 32581613 10.2147/RMHP.S232114 PMC7280239

[2] Bech M. The economics of non-attendance and the expected effect of charging a fine on non-attendees. Health Policy. 2005;74(2):181–91. 16153478 10.1016/j.healthpol.2005.01.001

[3] Weinger K, McMurrich SJ, Yi JP, Lin S, Rodriguez M. Psychological characteristics of frequent short-notice cancellers of diabetes medical and education appointments. Diabetes Care. 2005;28(7):1791–3. 15983337 10.2337/diacare.28.7.1791 PMC1584304

[4] Goldman K, Aldosari MA, Discepolo K. Missed dental care appointments in an urban safety net hospital. J Calif Dent Assoc. 2022;50(8):473–9.

[5] Muñoz-Pino N, Vives-Cases C, Agudelo-Suárez AA, Ronda-Pérez E. Comparing oral health services use in the Spanish and immigrant working population. J Immigr Minor Health. 2018;20(4):809–15. 28735453 10.1007/s10903-017-0630-4

[6] Hiilamo A, Keski-Säntti M, Mannevaara M, Kallio J, Harjunmaa U, Koskenvuo K. Toimeentulotukea Saaneiden Perheiden Lapsilla on Ilmoittamattomia poisjääntejä hammashoidosta Ja Hammaskariesvaurioita Muita useammin – rekisteritutkimus Vuonna 1997 syntyneistä Espoolaisista. 2023. https://ideas.repec.org/p/osf/socarx/ktfw9.html .

[7] Skaret E, Raadal M, Kvale G, Berg E. Missed and cancelled appointments among 12-18-year-olds in the Norwegian public dental service. Eur J Oral Sci. 1998;106(6):1006–12. 9879912 10.1046/j.0909-8836.1998.eos106605.x

[8] Skaret E, Raadal M, Kvale G, Berg E. Factors related to missed and cancelled dental appointments among adolescents in Norway. Eur J Oral Sci. 2000;108(3):175–83. 10872986 10.1034/j.1600-0722.2000.108003175.x

[9] Wang NJ, Aspelund GO. Children who break dental appointments. Eur Arch Paediatr Dent. 2009;0(1):11–4. 10.1007/BF03262660 19254520

[10] Åstrøm AN, Smith ORF, Sulo G. Early-life course factors and oral health among young Norwegian adults. Community Dent Oral Epidemiol. 2021;49(1):55–62. 32918289 10.1111/cdoe.12576

[11] Fägerstad A, Lundgren J, Windahl J, Arnrup K. Dental avoidance among adolescents - a retrospective case -control study based on dental records in the public dental service in a Swedish county. Acta Odontol Scand. 2019;77(1):1–8. 30022701 10.1080/00016357.2018.1489978

[12] Suominen-Taipale AL, Widström E, Sund R. Association of examination rates with children’s National caries indices in Finland. Open Dent J. 2009;3:59–67. 19543545 10.2174/1874210600903010059 PMC2697058

[13] Finnish Institute for Health and Welfare, Finland - THL. Suun ja hampaiden sairauksien hoidon laaturekisteri – tulosraportti. 2023. https://repo.thl.fi/sites/laaturekisterit/suun_terveyden_rekisteri/nqrodh.html#jump_leader2 .

[14] Finlex. Government Decree 338. /2011. Maternity and child health clinic services, school and student health services and preventive oral health services for children and youth. 2011. https://www.finlex.fi/en/laki/kaannokset/2011/en20110338.pdf .

[15] Vehkalahti M, Tarkkonen L, Varsio S, Heikkilä P. Decrease in and polarization of dental caries occurrence among child and youth populations, 1976–1993. Caries Res. 1997;31(3):161–5. 9165184 10.1159/000262392

[16] Linden J, Widström E, Sinkkonen J. Children and adolescents´ dental treatment in 2001–2013 in the Finnish public dental service. BMC Oral Health. 2019;19(1):131. 31262298 10.1186/s12903-019-0828-z PMC6604139

[17] Nihtilä A, Widström E. Heavy use of dental services among Finnish children and adolescents. Eur J Paediatr Dent. 2009;10(1):7–12. 19364239

[18] City of Helsinki. Health and social services. Dent Care. 2024. https://www.hel.fi/en/health-and-social-services/health-care/dental-care .

[19] Mensonen I, Goswami S, Kaila M, Tseveenjav B. No-shows in specialized dental care for children and adolescents in HUS. Suomen Hammaslääkärilehti (Finnish Dent Journal). 2025;5:38–43.

[20] Goswami S, Tseveenjav B, Kaila M. Non-utilization of oral health services and associated factors among children and adolescents: an integrative review. Acta Odontol Scand. 2023;81(2):105–18. 35841154 10.1080/00016357.2022.2095020

[21] Sun BC, Chi DL, Schwarz E, Milgrom P, Yagapen A, Malveau S, Chen Z, Chan B, Danner S, Owen E, et al. Emergency department visits for nontraumatic dental problems: a mixed-methods study. Am J Public Health. 2015;105(5):947–55. 25790415 10.2105/AJPH.2014.302398 PMC4386544

[22] Wigen TI, Skaret E, Wang NJ. Dental avoidance behaviour in parent and child as risk indicators for caries in 5-year-old children. Int J Paediatr Dent. 2009;19(6):431–7. 19708863 10.1111/j.1365-263X.2009.01014.x

[23] Wogelius P, Poulsen S. Associations between dental anxiety, dental treatment due to toothache, and missed dental appointments among six to eight-year-old Danish children: a cross-sectional study. Acta Odontol Scand. 2005;63(3):179–82. 16191913 10.1080/00016350510019829

[24] Iben P, Kanellis MJ, Warren J. Appointment-keeping behavior of Medicaid-enrolled pediatric dental patients in Eastern Iowa. Pediatr Dent. 2000;22(4):325–9. 10969442

[25] Tandon S, Duhan R, Sharma M, Vasudeva S. Between the cup and the lip: missed dental appointments. J Clin Diagn Res. 2016;10(5):Zc122–124. 27437344 10.7860/JCDR/2016/17400.7842 PMC4948520

[26] Gomes MAG, Abreu M, Ferreira FM, Fraiz FC, Menezes J. No-shows at public secondary dental care for pediatric patients: a cross-sectional study in a large Brazilian city. Cienc Saude Coletiva. 2019;24(5):1915–23. 10.1590/1413-81232018245.19312017 31166524

[27] Kirby J, Harris JC. Development and evaluation of a ‘was not brought’ pathway: a team approach to managing children’s missed dental appointments. Br Dent J. 2019;227(4):291–7. 31444446 10.1038/s41415-019-0621-z

[28] Fägerstad A, Windahl J, Arnrup K. Understanding avoidance and non-attendance among adolescents in dental care - an integrative review. Community Dent Health. 2016;33(3):195–207. 28509515 10.1922/CDH_3829Fagerstad13

[29] Stokes E, Ashcroft A, Platt MJ. Determining Liverpool adolescents’ beliefs and attitudes in relation to oral health. Health Educ Res. 2006;21(2):192–205. 16192312 10.1093/her/cyh055

[30] American Academy of Pediatric Dentistry. Adolescent oral health care. In: The Reference Manual of Pediatric Dentistry. Chicago, III: American Academy of Pediatric Dentistry; 2023. p. 317–26.

[31] Coolidge T, Heima M, Johnson EK, Weinstein P. The dental neglect scale in adolescents. BMC Oral Health. 2009;9(1):2. 19123953 10.1186/1472-6831-9-2 PMC2627830

[32] Davoglio RS, Abegg C, Aerts DR. Factors related to the use of dental services among adolescents from Gravataí, RS, Brazil, in 2005. Rev Bras Epidemiol. 2013;16(2):546–54. 24142024 10.1590/S1415-790X2013000200028

[33] Zimmer-Gembeck MJ, Alexander T, Nystrom RJ. Adolescents report their need for and use of health care services. J Adolesc Health. 1997;21(6):388–99. 9401858 10.1016/S1054-139X(97)00167-5

[34] Lebrun-Harris LA, Canto MT, Vodicka P. Preventive oral health care use and oral health status among US children: 2016 National survey of children’s health. J Am Dent Assoc. 2019;150(4):246–58. 30922456 10.1016/j.adaj.2018.11.023

[35] Ghanbarzadegan A, Bastani P, Luzzi L, Brennan D. Inequalities in utilization and provision of dental services: a scoping review. Syst Rev. 2021;10(1):222. 34376247 10.1186/s13643-021-01779-2 PMC8356458

[36] da Fonseca MA, Avenetti D. Social determinants of pediatric oral health. Dent Clin North Am. 2017;61(3):519–32. 28577634 10.1016/j.cden.2017.02.002

[37] Como DH, Stein Duker LI, Polido JC, Cermak SA. The persistence of oral health disparities for African American children: a scoping review. Int J Environ Res Public Health. 2019. 10.3390/ijerph16050710. 30818846 10.3390/ijerph16050710 PMC6427601

[38] Discepolo K, Melvin P, Ghazarians M, Tennermann N, Ward VL. Socioeconomic and clinical demography of dental missed care opportunities. JDR Clin Transl Res. 2023;8(4):356–66. 10.1177/23800844221104790 35722931

[39] Armfield JM, Heaton LJ. Management of fear and anxiety in the dental clinic: a review. Aust Dent J. 2013;58(4):390–407. 24320894 10.1111/adj.12118

[40] Rantavuori K, Lahti S, Hausen H, Seppä L, Kärkkäinen S. Dental fear and oral health and family characteristics of Finnish children. Acta Odontol Scand. 2004;62(4):207–13. 15513417 10.1080/00016350410001586

[41] Anagha KA, Megha M, Karuveettil V, Vijay Kumar S. Perceptions of barriers towards dental appointment keeping among patients of a tertiary care setting: A mixed method exploration. J Oral Biol Craniofac Res. 2024;14(2):185–19. 38405603 10.1016/j.jobcr.2024.02.002 PMC10891327

[42] Kaplan-Lewis E, Percac-Lima S. No-Show to primary care appointments:why patients do not come. J Prim Care Community Health. 2013;4(4):251–5. 24327664 10.1177/2150131913498513

